# Evaluation of *Roholtiella* sp. Extract on Bell Pepper (*Capsicum annuum* L.) Yield and Quality in a Hydroponic Greenhouse System

**DOI:** 10.3389/fpls.2022.843465

**Published:** 2022-07-05

**Authors:** Adewale Suraj Bello, Imen Saadaoui, Talaat Ahmed, Helmi Hamdi, Maroua Cherif, Radhouane Ben-Hamadou

**Affiliations:** ^1^Envrionmental Science Program, Department of Biological and Environmental Sciences, College of Arts and Sciences, Qatar University, Doha, Qatar; ^2^Algal Technologies Program (ATP), Center for Sustainable Development, College of Arts and Sciences, Qatar University, Doha, Qatar; ^3^Environmental Science Centre, Qatar University, Doha, Qatar; ^4^Food and Water Security Program, Center for Sustainable Development, College of Arts and Sciences, Qatar University, Doha, Qatar

**Keywords:** high-value product extract, water resuspended biomass, bell pepper (*Capsicum annuum* L.), ascorbic acid, phenolic compound, hydroponic systems

## Abstract

This study was carried out to investigate the impacts of cyanobacteria (*Roholtiella* sp.) high-value product extract (HVPE) and water resuspended biomass WRB treatments on bell pepper production using the hydroponic system under greenhouse conditions. Six cyanobacteria treatments (6 ml L^−1^, 4 ml L^−1^, and 2 ml L^−1^ – HVPE, 6 ml L^−1^, 4 ml L^−1^, and 2 ml L^−1^ – WRB, and TR0 as control) were evaluated using the foliar application method. The results showed that foliar application of HVPE with treatments of 2 ml L^−1^, 4 ml L^−1^, and 6 ml L^−1^ produced significantly higher values of physical growth parameters of bell pepper (BP) plants (shoot length, the number of leaves, plant leaf length, plant leaf width, and the diameter of the shoot), SPAD index, yield components (the fruit length, fruit width, the number of fruit per plant, and fresh weight per fruit), biochemical composition [ascorbic acid, phenolic acid, and total soluble solids (TSS)], and the total yield compared to the control group TR0. Also, significant higher values of growth parameters (shoot length, the number of leaves, plant leaf length, plant leaf width, the diameter of the shoot), SPAD index, yield components (the fruit length, fruit width, the number of fruits per plant, and fresh weight per fruit), biochemical composition [ascorbic acid, phenolic acid, and total soluble solids (TSS)], and the total yield were obtained with foliar spraying WRB at 2 ml L^−1^, 4 ml L^−1^, and 6 ml L^−1^ compared to the control group TR0. Consequently, the treated bell pepper with *Roholtiella* sp. HVPE and WRB were more efficient in enhancing production and chemical constituents compared with the control group.

## Introduction

The persistent rise in global industrialization, urbanization, and technological advancement is among the reasons why the global population is on the rise; it is forecasted that the population is expected to exceed 9.7 billion by 2050 (Abdelaal et al., 2020). Thus, the population increase coupled with the ecological implications of climatic change makes agriculture liable to several constraints such as abiotic stresses, causing negative impacts on crop production globally (Soliman et al., 2018; Mutale-joan et al., 2021). Generally, the conventional methods of agriculture, namely, irrigation farming, mechanized farming, and application of chemical fertilizers (e.g., urea and NPK) to maximize plant productivity and the attainment of the optimum plant's growing conditions are used in practice (Elkeilsh et al., 2019). However, these conventional techniques have been practiced for decades but are seen to pose some threat to the environment and the existing ecosystem as a whole (Carvalho, 2017; Rahman and Zhang, 2018). Consequently, to reduce these effects, there is a need for practicing modern agriculture as an alternative eco-friendly and sustainable technique. The discovery of green technologies that make use of bioresources such as biofertilizers and biostimulants is widely accepted as innovative options to improve crop growth and productivity (Calvo et al., 2014; Elzaawely et al., 2017; Van Oosten et al., 2017; Yakhin et al., 2017; Desoky et al., 2018; Bello et al., 2021a,b).

Bell pepper (BP) (*Capsicum annuum* L.) is an essential vegetable that belongs to the Solanaceae family. It remains one of the most important and universally cultivated vegetables globally owing to its high economic and multiple health benefits (Abdelaal et al., 2020). The bell pepper is unarguably one of the richest vegetables in ascorbic acid content (Rao et al., 2011; Domínguez-Martínez et al., 2014; Endo et al., 2019; Papathanasiou et al., 2021; Rehman et al., 2021). Interestingly, it has been established that one fruit of a bell pepper weighing 70 g or more can supply the daily necessary amount of vitamin C required for metabolic activity by humans (Marhoon and Abbas, 2015). Also, a good reasonable amount of essential vitamins A, B1, and other vitamins suitable for growth are embedded in bell pepper (Marhoon and Abbas, 2015; Awad-Allah et al., 2021; Mohamed et al., 2021). Synthetic chemical fertilizer and most inorganic commercial hydroponic nutrients are among the most productive and effective practices to manage crop production for a better yield. However, their potential adverse effects on the environment coupled with a decrease in their efficiency after excessive and continuous applications have necessitated the need to restrict or drastically limit the use of chemical fertilizers and inorganic nutrients to come up with better, sustainable, and eco-friendly alternatives. Thus, organic fertilizers and eco-friendly practices for crop production and management, including fertigation and pest control, are the best options (Nair et al., 2020; Shah et al., 2021). The blue-green algae (BGA), i.e., cyanobacteria, form essential constituents of the soil microflora that increase soil nutrient composition and productivity either directly or indirectly (Vaishampayan et al., 2001; Mishra and Pabbi, 2004; Shariatmadari et al., 2015). Different research studies have shown that the application of microalgae and cyanobacteria either as biomass (particle form) or extract (liquid form) contributes immensely in increasing the nitrogen composition of the soil as well as growth-enhancing substances such as phytohormones, amino acids, phenolic compounds, and important trace elements found to be essential for plant health, development, and ion distribution (Obana et al., 2007; Cuellar-Bermudez et al., 2015; de Morais et al., 2015; Shariatmadari et al., 2015; Renuka et al., 2018).

Also, the use of cyanobacterial extracts as biofertilizers through foliar application will supply organic and natural plant hormones, namely, auxin, auxin compounds, cytokinins, vitamins, and numerous macro and microelements, that enhance plant development (El-Eslamboly et al., 2019). Naturally, cyanobacterial extract and biomass are seen as one of the novel sources used to attain sustainability and increase agricultural production, respectively, by gradually shifting away from using inorganic chemical fertilizers that are characterized by their numerous environmental issues. Furthermore, many studies have established the importance of cyanobacteria to improve the release of essential metabolites in plants (Saker et al., 2000; Shanab, 2001). Interestingly, the positive effects of microalgae and cyanobacteria extracts and biomass have been widely studied by numerous researchers/scholars as biostimulants and biofertilizers suitable for the production crops, including rice, maize, and wheat (Barone et al., 2018; Ronga et al., 2019; Carillo et al., 2020; Colla and Rouphael, 2020), but more attention needs to be paid to the application and effect of cyanobacteria on vegetables, particularly bell pepper, considering its benefits to humans.

This study aims to investigate the effect of *Roholtiella* sp. as a bionutrient on bell pepper growth performance as well as on fruit yield and quality, including nutrient composition, under a greenhouse large-scale hydroponic production system.

## Materials and Methods

### Plant Material

Bell pepper (*Capsicum annuum* L.) hybrid “Naisa” (Seminis 11846215/100151N1-1080) seeds were first planted in the germination trays of seven by 12 cells making 84 cells. Each of the cells was filled with pot soil to raise the seedlings. They were irrigated with a commercial nutrient solution. The 30-day-old seedlings were transplanted to a greenhouse under a hydroponic system (drip technique). The agricultural practices, such as weeding, pruning, pest management, and fertigation, conformed with the commercially standardized practices and were executed as recommended by the local authorities for bell pepper cultivation.

### Cyanobacteria Strains' Cultivation and Growth Conditions

*Roholtiella* sp. (QUCCC97) was obtained from Qatar University's Culture Collection of Cyanobacterium and Microalgae (QUCCCM), Doha, Qatar, and was originally isolated from Qatar soil (Saadaoui et al., 2016). The selection of the strain was based on its positive impact on the seedlings of bell pepper and its pigment composition based on a previous study (Bello et al., 2021a). One single colony of the cyanobacteria strain was used to inoculate a 5 ml volume of BG11 growth medium (Stanier et al., 1971). It was subsequently incubated for 12 days at 30°C under the following conditions: a photon flux density of 100 μmol photons m^−2^ s^−1^ and a 12:12 h dark:light cycle with 150 rpm agitation using an illuminated shaker (Innova 44R, New Brunswick Scientific, USA). Then, the culture was gradually scaled up to 500 ml and incubated under the previously described conditions. Then, this culture was scaled up to 2 L then 10 L under room temperature, air bubbling, and the illumination of 400 μmol photons m^−2^ s^−1^ before being used to inoculate an open raceway pond of 200 L capacity. This pilot-scale cultivation of *Roholtiella* sp. was performed outdoors in Qatar's climate. Environmental parameters from winter to spring were recorded for the duration of the experiment. After 12 days of outdoor incubation, the biomass was harvested by centrifugation and then freeze-dried. Thus, the total time required for cyanobacteria culture and scale-up to generate the required biomass for the bioassay was 72 days, and the culture was performed in duplicate.

### Preparation of Cyanobacteria (*Roholtiella* sp.) Extract

The preparation of cyanobacterial extract followed the method described previously by Bello et al. (2021a). In brief, the freeze-dried biomass obtained after the cultivation explained earlier was divided equally into two portions. One part was resuspended in water (WRB), which was used directly as a biofertilizer, and the second part underwent aqueous extraction to obtain the phycobiliproteins, a high-value product extract (HVPE). The methodology has been fully described in our earlier study conducted on the efficacy of cyanobacteria extract and biomass on bell pepper production (Bello et al., 2021a).

### Analysis of Chemical Compositions of Cyanobacteria Extract and Biomass

In our previous study, the cyanobacterium *Roholtiella* sp. was characterized morphologically, also through optical measurement of pigments and molecular identification (Bello et al., 2021a). The chemical composition of this cyanobacterium (*Roholtiella* sp.) HVPE and WRB were analyzed using the ion chromatography method at the Central Laboratory Unit, Qatar University, Qatar. The analyzed chemicals include Sodium (Na^+^), Ammonium (NH${}_{4}^{+}$), Potassium (K^+^), Calcium (Ca^++^), Magnesium (Mg^++^), Fluoride (F^−^), Chloride (Cl^−^), Nitrate (NO${}_{3}^{-}$), Phosphate (PO${}_{4}^{--}$), Sulfate (SO${}_{4}^{-}$). This is considered to be necessary to further confirm the presence of a significant amount of mineral nutrients in both HVPE and WRB.

### Greenhouse Hydroponics Experiment and Growth Conditions

The experiment was carried out for ~180 days from seed germination to fruiting/harvesting. The seedlings were transplanted into white grow pots (25 × 25 × 30 cm) at one seedling per pot a month after germination. The grow pot is filled with cocopeat as the substrate for plant support and provision of structure for root development. The temperature was maintained at a range of 21–26°C, respectively, in the greenhouse at Al Sulaiteen Agricultural & Industrial Complex, Umm Salal Ali, Qatar. The greenhouse hydroponic system is a drip technique that is fully automated with the adoption of a cooling system. The experiment was conducted between February and August 2021. Thus, healthy seedlings with similar physical appearances and relatively the same weight were considered for the propagation to commence the experiment and to ensure uniformity.

### Treatments

Seven treatments were executed during the growing period. HVPE-1, HVPE-2, and HVPE-3 (foliar application with *Roholtiella* sp. HVPE), WRB-1, WRB-2 and WRB-3 (foliar application with *Roholtiella* sp. WRB), and Tr0 (foliar application with water as a control). Foliar spraying was done every week at the rate of 0.576 ml stroke^−1^ leaf^−1^ and thus, increased to two strokes as the leaf area increased, which lasted for 6 weeks as summarized in Table 1.

**Table 1:** The concentrations of *Roholtiella* sp. extracts and biomass.

**Strain**	**Treatments/concentration**
HVPE from *Roholtiella* sp.	Tr1— (2 ml L^−1^)−0.2% concentration (2 ml HVPE of *Roholtiella* sp.)
	Tr2— (4 ml L^−1^)−0.4% concentration (4 ml HVPE of *Roholtiella* sp.)
	Tr3— (6 ml L^−1^)−0.6% concentration (6 ml HVPE of *Roholtiella* sp.)
WRB from *Roholtiella* sp.	Tr4— (2 ml L^−1^)−0.2% concentration (2 ml WRB of *Roholtiella* sp.)
	Tr5— (4 ml L^−1^)−0.4% concentration (4 ml WRB of *Roholtiella* sp.)
	Tr6— (6 ml L^−1^)−0.6% concentration (6 ml WRB of *Roholtiella* sp.)
Control	Tr0- water

### Parameters and Measurements

There are seven blocks/replicates, seven plots per block, and five plants per plot. Therefore, we have 35 plants per block. Each of the 7 treatments (2, 4, and 6 ml for HVPE; 2, 4, and 6 ml for WRB + control = 7 treatments) was applied per plot. A total of 147 plants were considered for the vegetative parameters. For the leaf length and width, 3 leaves are randomly selected and an average taken. Physical growth parameters were recorded at different times after transplanting. Randomly selected plants in each plot were considered and evaluated as follows: plant height (cm), number of leaves per plant, plant leaf length (cm), plant leaf width (cm), the diameter of the shoot, and SPAD index.

#### Plant Height (cm) – SL

The plant height of bell pepper was taken from the top of the support, assumed to be the base, to the topmost part where the leaves are fully opened by using a measuring scale, and then the average was recorded. The measurements were carried out at the commencement and completion of the experiment, respectively.

#### Number of Leaves per Plant – NL

The number of leaves per plant was counted manually 20 days after the seedlings were transplanted.

#### Plant Leaf Length and Width (cm)

The length of the leaf and its width were measured using a measuring ruler. Fully expanded leaves were considered, and the measurement runs from the base of the leaf to the tip. The average length and width were taken and later entered into the spreadsheet.

#### Stem Diameter (cm)

The width of the stem was taken to determine the diameter of the plants. Stem width was measured (cm) manually with an electronic digital vernier caliper (Clarke CM145 Digital Vernier Caliper) at the 6th and 8th weeks after transplanting.

#### SPAD Index – SI

The SPAD index of bell pepper leaf was measured using SPAD-02, a chlorophyll meter (Konica Minolta Optics, Osaka, Japan). The SPAD index measurement was carried out before the harvest commences/termination of the experiment in the second week of the experiment when the leaves were very green and healthy.

### Fruit Parameters

#### Length of Fruit (cm) and Diameter/Width of Fruit (cm)

The length and width (cm) of the fruit were manually determined using an electronic digital vernier caliper (Clarke CM145 Digital Vernier Caliper) at every harvest for 12 weeks the harvest lasted, and the values were recorded at all times for further use.

### Output and Its Constituents

The output (yield) and its constituents are considered as follows:

#### Total Yield per Plant

The total weight of the fruits harvested was determined at the end of the experiment at the 12th week of cultivation using a measuring standard laboratory scale, which was the summation of every harvest for the entire harvesting period. The summation of the weekly harvest was recorded in grams per fruit, and this amounted to the total yield per plant.

#### Total Number of Fruits per Plant

The number of fruits collected per plant at every harvest (the weekly number of fruits per plant) was manually counted and recorded in a spreadsheet. After the cultivation, the summation of the recorded values (the weekly number of fruits per plant) was obtained as the total number of fruits per plant obtained based on the various treatments.

#### Average Fruit Weight (g) per Plan

The average weight of fruit per plant is computed by dividing the total weight of fruits (g) by the total number of fruits per plant.


$$\begin{array}{l} \text{Average fruit weight }\left(\text{g}\right)=\;\frac{\text{Total weight }\left(\text{g}\right)\text{ of fruits per plant}}{\text{Total number of fruits per plant}} \end{array}$$

#### Total Yield (Kg/Treatment)

The summation of the total yield of fruits per plant per treatment was recorded as the total yield in grams and converted to a kilogram per treatment.

### Chemical Component Analyses/Fruit Nutritional Value

#### Total Soluble Solids (TSS in °Brix)

Total soluble solids is regarded as an index of soluble solid concentration in the fruit. Thus, each whole bell pepper was ground using a Kenwood blender/mixer (Blend-X Compact Blender) until a homogeneous mixture was produced. Thereafter, about 45 g of subsample was extracted and centrifuged for 20 min at 1,700 × *g*. To determine the TSS, the filtrate was then taken and placed on the handheld digital refractometer (ATAGO, USA Inc., Kirkland, WA, USA). Generally, the results were expressed as Brix (AOAC International, 2016; Continella et al., 2018; Cortés-Estrada et al., 2020).

#### Ascorbic Acid Determination (Xylene Assay)

Ascorbic acid (Vitamin C) was extracted through the xylene extraction method (Ranganna, 1986). The concentration was estimated from the absorbance measured at 520 nm using a Jenway 6715 UV/Visible Scanning Spectrophotometer, 1.5-nm Bandwidth.

Standard curve: Six test tubes were prepared with an ascorbic standard solution of 0.0, 0.5, 0.75, 1, 1.5, and 2 ml in 3% orthophosphoric acid H_3_PO_3_ (100 μg ml^−1^) and makeup to 2 ml of 3% H_3_PO_3_ solution. Thereafter, 2 ml of acetate buffer (pH 4) was added, followed by adding 3 ml of 3,6-dichlorophenol indophenol reagent, and 15 ml of xylene solution simultaneously in quick succession. The capped tubes were vortexed for 10–15 s to create phase separation. The xylene topmost phase was extracted and absorbance was measured spectrophotometrically at a wavelength of 520 nm using xylene as a blank (Domínguez-Martínez et al., 2014).

Sample analysis/extraction procedure: Bell pepper (100 g) was macerated in 3% H_3_PO_3_ and the volume was made up to 200 ml. Followed by the filtration of the solution, transferring 2 ml of aliquot to a test tube, adding one after the other: 2 ml of acetate buffer (pH 4), 3 ml of 2,6-dichlorophenol indophenol (7 × 10^−4^ M), and 15 ml xylene in quick succession. Thereafter, the capped tube was vortexed for 10–15 s. The topmost xylene phase was extracted, and absorbance was taken at 520 nm. The sample absorbance was read against the blank prepared in a similar way above but substituting 2,6-dichlorophenol indophenol with distilled water instead. The ascorbic acid content was denoted as mg acid 100 g^−1^ sample (Deepa et al., 2007; Beltrán-Orozco et al., 2009).

#### Total Phenolic Compounds

Standard curve: Solutions containing 0, 20, 40, 60, 80, and 100 mg L^−1^ gallic acid standard were prepared respectively. In total, 0.1 ml was transferred to each of the test tubes, adding 0.1 ml deionized water, 1 ml Folin-Ciocalteau reagent, and 0.8 ml of 7.5% soda crystals (Na_2_CO_3_). The tubes were vortexed, and the final solution was incubated for 30 min in the dark at room temperature. The absorbance of the solution was measured at 765 nm spectrophotometrically (Jenway 6715 UV/Visible Scanning Spectrophotometer, 1.5-nm Bandwidth) against a blank prepared in the same way but replacing Folin-Ciocalteau with distilled water (Domínguez-Martínez et al., 2014).

Sample analysis/extraction procedure: Bell pepper (2.0 g) was macerated in and extracted with 80% methanol, vortexed three times for 50–60 s, and then centrifuged for 15 min at 500 rpm. Thereafter, the final mixture was separated by simple decantation. The supernatant/solvent of the combined extract was concentrated in a flash evaporator to obtain a final volume of 10 ml (Domínguez-Martínez et al., 2014).

Determination of phenolic compound: To determine the total phenolic compound, 2 ml of the extract was pipetted into the test tube, adding 1 ml Folin-Ciocalteau reagent, 2 ml Na_2_CO_3_ (20%), and 2.5 ml distilled water. The final solution is incubated for 30 min in the dark at room temperature. Subsequently, the absorbance of the solution was measured at 765 nm spectrophotometrically (Jenway 6715 UV/Visible Scanning Spectrophotometer, 1.5-nm Bandwidth) against a blank prepared in the same way but substituting Folin-Ciocalteau with distilled water. The total phenolic compound was computed using the phenolic standard curve (*y* = *aX*+*C*). The results were presented in gallic acid equivalents (GAE) per gram of fresh weight (μg GAE g^−1^ fw).

### Experimental Design and Data Analysis

A randomized complete block design (RCBD) experiment's layout was put in place, with seven blocks. Each block is a replicate, and each replicate is equivalent to the seven plots at five plants per plot. The analysis of variance (ANOVA) was used to analyze the data using a statistical tool. Additionally, Tukey's Honest Significant Difference test was conducted to establish the range distribution as a post-doc test, thus comparing the means within and among treatments. Also, Pearson's correlation matrix was calculated to determine the interaction or relationship between plant vegetative growth factors, biochemical parameters, yield, and its components, phenolic compounds, and ascorbic acid contents of bell pepper cultivated in hydroponic systems under *Roholtiella* sp. HVPE and WRB treatment, respectively, as a growth enhancer/promoter (biofertilizer).

## Results

### Microalgae (Cyanobacteria, *Roholtiella* sp.)

#### Cyanobacteria Chemical Characterization

*Roholtiella* sp. is one of three promising cyanobacteria strains previously investigated with the aim of evaluating their growth-enhancing abilities (Bello et al., 2021a). Among them, *Roholtiella* sp. was selected for further analysis based on its better performance. However, the elemental composition of *Roholtiella* sp. HVPE and WRB evidence the availability of a considerable amount of both anions and cations, respectively, as shown in Table 2.

**Table 2:** Chemical content of cyanobacteria extract and biomass.

**Anions/**	**Analytical**	***Roholtiella***	***Roholtiella***
**Cations (ppm)**	**method**	**extract**	**biomass**
Sodiu	Ion chromatography	2.379	9.501
Ammonium (NH4^+^)		0.674	0.641
Potassium (K^+^)		8.533	0.541
Calcium (Ca^++^)		1.777	0.594
Magnesium (Mg^++^)		3.483	0.255
Fluoride (F^−^)		0.0191	0.02
Chloride (Cl^−^)		3.168	1.192
Nitrate (NO${}_{3}^{-}$)		5.247	8.071
Phosphate (PO${}_{4}^{3-}$)		13.67	3.266
Sulfate (SO${}_{4}^{2-}$)		0.212	0.12

### Impacts of *Rohotiella* sp. HVPE and WRB Treatments on the Biometric/Vegetative/Physical Growth Parameters and SPAD Index

**Figure 1:**
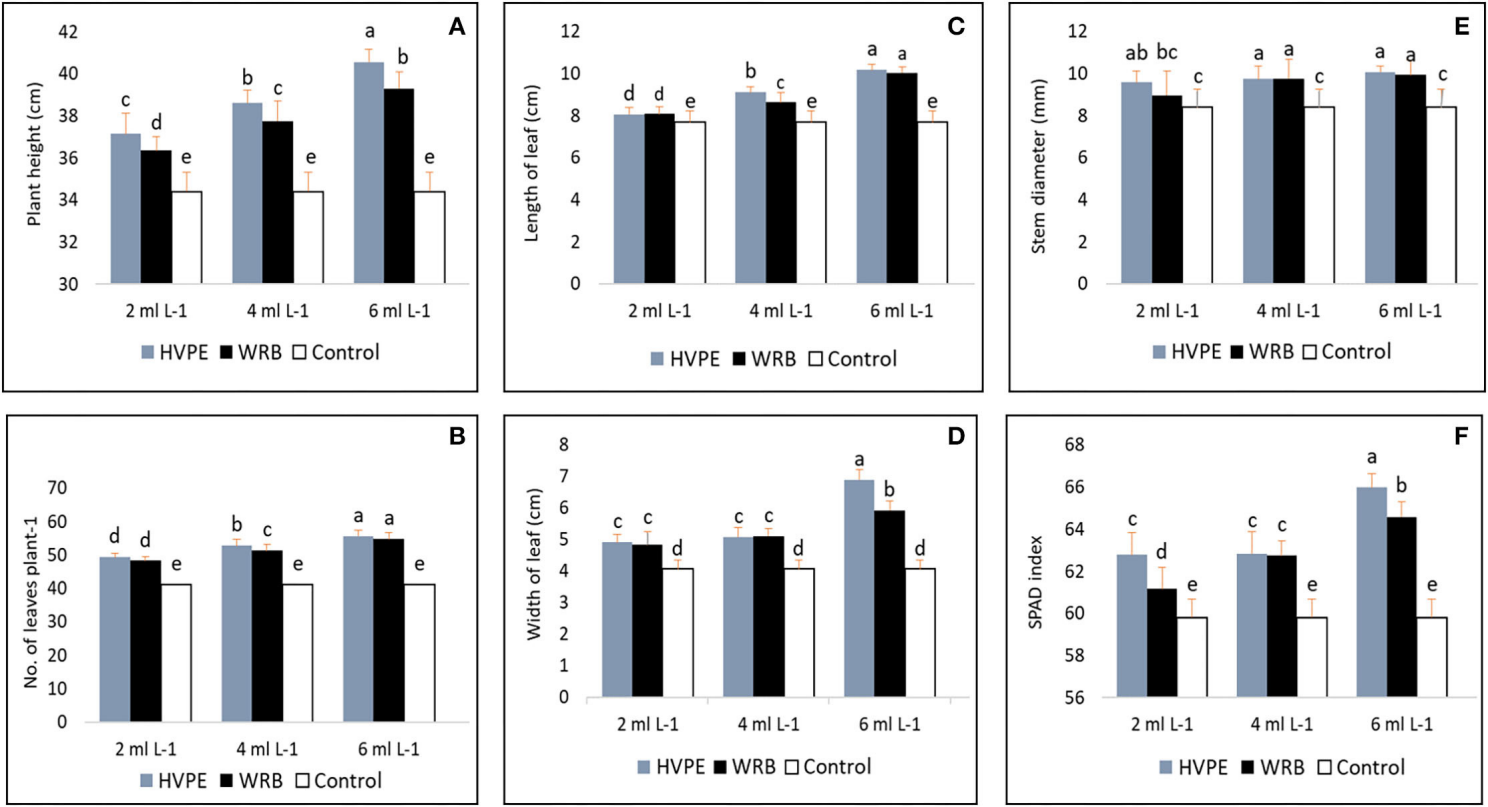
Plant height, number of leaves, length of leaf, the width of leaf, stem diameter, and spad index **(A–F)** of bell pepper untreated plants (Tr0), treated with 2 mL L^−1^ (HVPE-1), 4 mL L^−1^ (HVPE-2), 6 mL L^−1^ (HVPE-3) of the HVPE. Additionally, with 2 mL L^−1^ (WRB-1), 4 mL L^−1^ (WRB-2), 6 mL L^−1^ (WRB-3) of the WRB. Plant height **(A)**, Number of leaves plant^−1^ **(B)**, Length of the leaf **(C)**, Width of the leaf **(D)**, Stem Diameter **(E)**, SPAD Index **(F)**.

As shown in Figures 1A–F, the bell pepper plant height, the number of leaves, stem diameter, and SPAD index are significantly increased compared to the characteristics of the control group in the cultivation period/grown season. All the treatments applied exhibited a significant difference in all the vegetative parameters and SPAD index. The *Roholtiella* sp. HVPE at 6 ml L^−1^ (HVPE-3) recorded the highest plant height with an increase of 15.15%. Also, bell peppers treated with 4 ml L^−1^ (HVPE-2) and 2 ml L^−1^ (HVPE-1) HVPE concentrations showed increased plant height by 10.93 and 7.37%, respectively, compared with the control group (TR0). Similarly, the various concentrations of WRB, 6 ml L^−1^ (WRB-3), 4 ml L^−1^ (WRB-2), and 2 ml L^−1^ (WRB-1) exhibited a significant increase (*p* > 0.05) in shoot length by 12.43, 8.87, and 5.38%, respectively, when compared to the control. Furthermore, HVPE treatments HVPE-3, HVPE-2, and HVPE-1 exhibited a significant increase in the number of leaves by 26.09, 22.16, and 16.68%, respectively, when compared to the control groups. In the same vein, WTB concentrations of WRB-3, WRB-2, and WRB-1 showed a significant increase (*p* > 0.05) in the number of leaves by 25.00, 19.78, and 14.79%, respectively, when compared with TR0. As compared to TR0, *Roholtiella* sp. HVPE and WRB exhibited a significant increase in the leaf length and width, respectively. Bell peppers treated with HVPE-3, HVPE-2, and HVPE-1 HVPE concentrations showed a significant increase in plant leaf length by 24.36, 15.58, and 4.66% and with WBR-3, WRB-2, and WRB-1 of the WRB by 23.36, 11.02, and 4.83%, respectively. Similarly, treatments with HVPE-3, HVPE-2, and HVPE-1 HVPE concentrations showed a significant increase in plant leaf width by 41.12, 20.00, and 17.20%, and for WRB treatments at the same concentrations, WBR-3, WRB-2, and WRB-1 by 31.40, 20.45, and 15.98%, respectively. Also, HVPE treatments followed the same trend by showing a significant increase in the diameter of the shoot by 16.49, 13.72, and 12.29% and a SPAD index by 9.36, 4.84, and 4.73%, respectively. Similarly, the diameter of the shoot increased by 15.33, 13.90, and 6.21% and a SPAD index by 7.40, 4.69, and 2.22%, respectively, when compared with TR0.

### Impacts of *Rohotiella* sp. HVPE and WRB Treatments on the Fruit Characteristics

**Figure 2:**
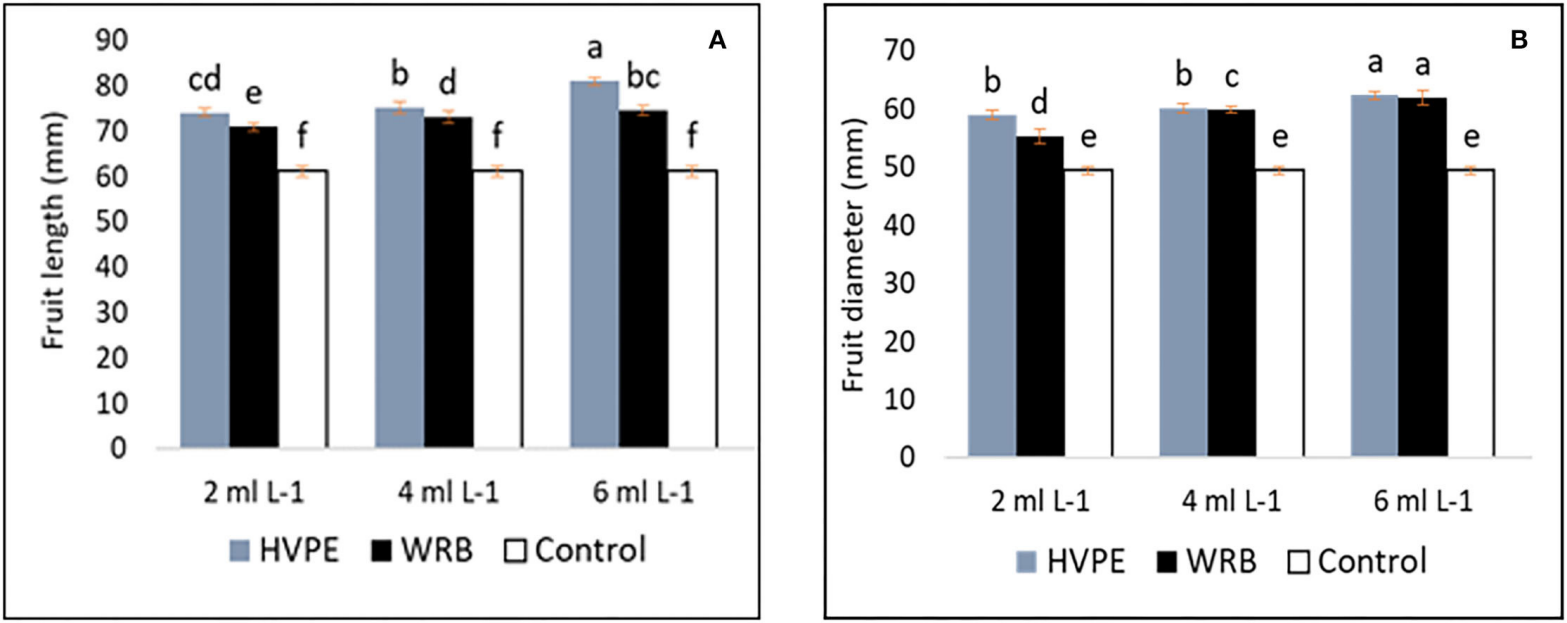
Fruit length and width (diameter) **(A,B)** of bell pepper untreated plants (Tr0), treated with 2 mL L^−1^ (HVPE-1), 4 mL L^−1^ (HVPE-2), 6 mL L^−1^ (HVPE-3) of the HVPE. Additionally, with 2 mL L^−1^ ((WRB-1), 4 mL L^−1^ (WRB-2), 6 mL L^−1^ (WRB-3) of the WRB. Fruit Length **(A)** and Fruit Diameter **(B)**.

Figures 2A,B shows that the fruit length and width (diameter) of bell peppers had significant increases compared to the characteristics exhibited by the control group with all the treatments. However, the highest fruit length with HVPE treatment was observed at HVPE-3, HVPE-2, and HVPE-1, respectively, at an increase of 24.51, 18.62, and 17.43% when compared with the TR0. Interestingly, the trend was similar with the fruit width at the same concentrations at HVPE-3, HVPE-2, and HVPE-1 when compared with the TR0. The fruit width is thus increased by 13.08, 16.32, and 17.90%. Similarly, the fruit length and width increased (*p* > 0.05) by 16.21, 17.74, and 20.68% and 10.51, 17.50, and 20.27%, respectively, with WRB at the same concentrations (WBR-3, WRB-2, and WRB-1) as compared with the control group.

### Impacts of *Rohotiella* sp. HVPE and WRB Treatments on the Yield Components and Total Yield

**Figure 3:**
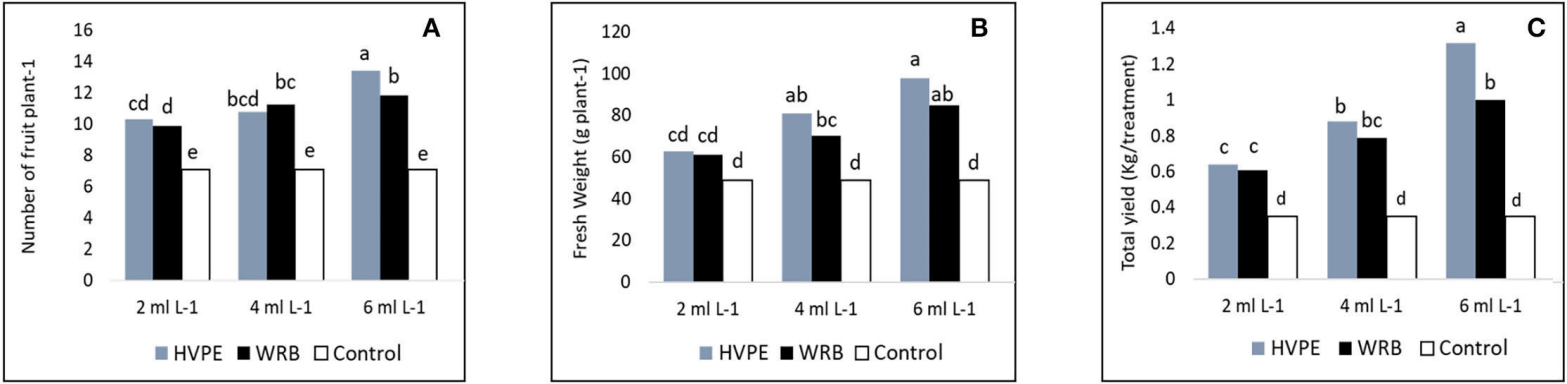
The number of fruits plant-1, fresh weight of fruit plant-1, and the total yield **(A–C)** of bell pepper untreated plants (Tr0), treated with 2 mL L^−1^ (HVPE-1), 4 mL L^−1^ (HVPE-2), 6 mL L^−1^ (HVPE-3) of the HVPE. Additionally, with 2 mL L^−1^ (WRB-1), 4 mL L^−1^ (WRB-2), 6 mL L^−1^ (WRB-3) of the WRB. Number of fruit plant -1 **(A)**; Fresh Weight **(B)**, and Total yield **(C)**.

It is clear from the obtained results as shown in Figure 3 (Castro-Puyana et al., 2017) that the number of fruits per plant, fresh weight of fruit per plant, and total yield were significantly increased at all levels of concentration of *Roholtiella* sp. HVPE and WRB when compared with the TR0. The number of fruits per plant increased significantly with the various concentrations of *Roholtiella* sp. HVPE when compared to the TR0. Thus, the concentrations of HVPE, HVPE-3, followed by HVPE-2, and HVPE-1 increased the number of fruits per plant by 47.17, 34.37, and 31.34%, respectively, compared to the TR0. Equally, these different concentrations of WRB (WBR-3, WRB-2, and WRB-1) caused significant increases (*p* > 0.05) in the number of fruits per plant by 40.16, 36.87, and 28.37%, when compared with the TR0.

At the same concentrations of *Roholtiella* sp. HVPE, the bell pepper showed a significant increase in the fruit weight per plant and total yield by 49.82, 39.23, 21.60, 73.35, 60.09, and 45.43%. The treatments showed a similar trend with WRB treatment at the same concentrations for the fruit weight per plant and total yield by 42.23, 30.25, and 19.82% and 64.98, 55.52, and 42.15% respectively, when compared with the TR0.

### Effect of *Rohotiella* sp. HVPE and WRB on Chemical Component Analyses/Fruit Nutritional Value

**Figure 4:**
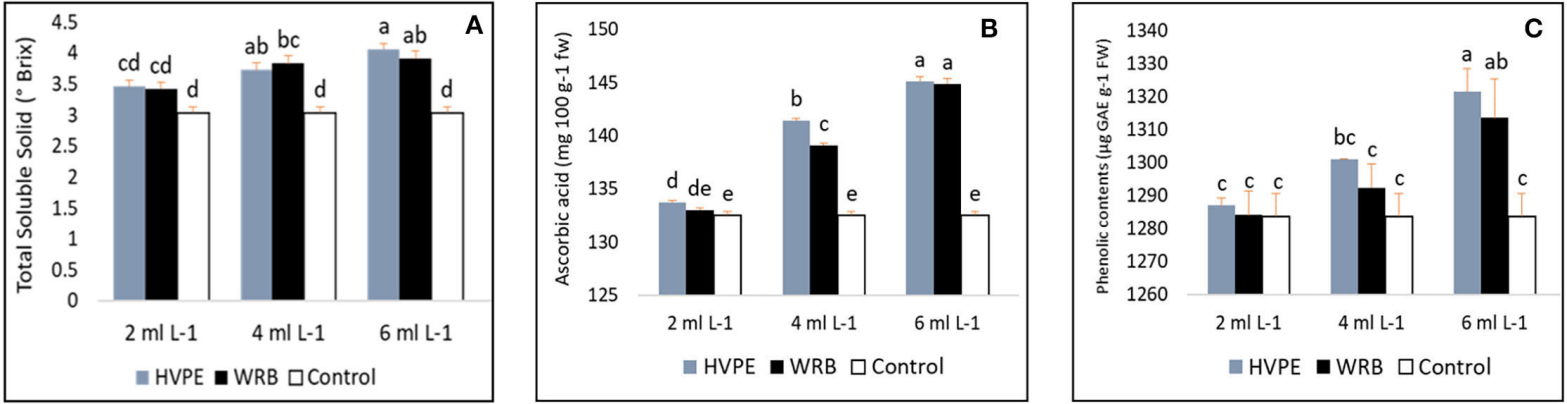
Total soluble solids, ascorbic acid, and the total phenolic content **(A–C)** of bell pepper untreated plants (Tr0), treated with 2 mL L^−1^ (HVPE-1), 4 mL L^−1^ (HVPE-2), 6 mL L^−1^ (HVPE-3) of the Roholtiella sp HVPE. Additionally, with 2 mL L^−1^ (WRB-1), 4 mL L^−1^ (WRB-2), 6 mL L-1 (WRB-3) of the Roholtiella sp WRB. Total Soluble Solid **(A)**; Ascorbic Acid **(B)**; Phenolic Content **(C)**.

As shown in Figures 4A–C, the fruit nutritional value, namely, the total soluble solids (TSS), ascorbic acid, and total phenolic acid, significantly increased with the treatments compared to the control group. From our results, the different concentrations of *Roholtiella* sp. HVPE (HVPE-1, HVPE-2, and HVPE-3) and WRB (WRB-1, WRB-2, and WRB-3) have a significant effect on the TSS content of bell pepper fruit, except at the minimum concentrations for both HVPE and WRB (HVPE-1 and WRB-1), where the change was not significant. The total soluble solids significantly increased with the various concentrations of *Roholtiella* sp. HVPE compared to the TR0. Consequently, the concentration of HVPE-3, followed by HVPE-2, and HVPE-1, respectively, increased (*p* > 0.05) the total soluble solids of fruits by 25.20% (4.06°Brix), 18.54% (3.728°Brix), and 12.20% (3.459°Brix) compared to the TR0 (3.037°Brix). The pattern is similar with the WRB treatment as TSS increased by 22.33% (3.91°Brix), 20.70% (3.83°Brix), and 11.33% (3.425°Brix) compared with the TR0 (3.037°Brix) as shown in Figure 4A.

#### Ascorbic Acid

From our results, as shown in Figure 4B, the ascorbic acid content in the fruits of bell pepper studies under different treatments indicates a significant difference from the TR0. The ascorbic content (vitamin c) in the fruits treated with HVPE-3 *Rohotiella* sp. HVPE is (145.10 mg 100 g^−1^ fw) per 100 mg fresh weight, accounts for a significant increase of 8.65% compared to the control group. Similarly, at the concentrations of HVPE-2 (141.42 mg 100 g^−1^ fw) and HVPE-1 (133.74 mg 100 g^−1^ fw), there is a significant increase (*p* > 0.05) in the ascorbic acid content by 6.27 and 0.89%, respectively, compared to the TR0 (132.55 mg 100 g^−1^ fw). In a similar manner, the trend followed the same pattern with the WRB treatments, with a significant increase of 8.53% with WRB-3 (144.90 mg 100 g^−1^ fw), 4.73% with WRB-2 (139.12 mg 100 g^−1^ fw), and 0.37% with WRB-1 (133.04 mg 100 g^−1^ fw) compared with the TR0 (132.55 mg 100 g^−1^ fw).

#### Total Phenolic Compounds

The findings on the total phenolic content of bell pepper are shown in Figure 4C. There is variation in the total phenolic concentration in the bell pepper as influenced by different treatments. At the highest concentration of HVPE-3 (1,321.23 μg GAE g^−1^ fw) of *Roholtiella* sp. HVPE, there is a significant increase (*p* > 0.05) of 2.85%, HVPE-2 (1,301 μg GAE g^−1^ fw) by 1.33% and TR1 (1286.96 μg GAE g^−1^ fw) by 0.26%, respectively. Furthermore, the trend is the same with treatments using WRB at the same concentration (1,313.46 μg GAE g^−1^ fw), (1,292.34 μg GAE g^−1^ fw), and (1,284.06 μg GAE g^−1^ fw) as the phenolic concentration increased by 2.27, 0.67, and 0.03% compared with the group control (1,283.64 μg GAE g^−1^ fw).

### Pearson's Correlation Studies

In this study, as shown in Table 3, chlorophyll content (SPAD index) showed a positive and significant correlation with shoot height (*r* = 0.954), number of leaves (*r* = 0.918), width of leaves (*r* = 0.892), and fruit weight (*r* = 0.892) as well. In a similar manner, the relationship trend between the growth vegetative parameter chlorophyll content and fruit weight per plant, as well as the total soluble solids, is positive and significantly correlated; fruit weight per plant (*r* = 0.991), and total soluble solids (*r* = 0.874) respectively. Also, a positive correlation was recorded between the phenolic compound and SH = shoot height (*r* = 0.983), NL = number of leaves (*r* = 0.981), SI = SPAD index (*r* = 0.946), and SD = shoot diameter (*r* = 0.82). Furthermore, the ascorbic acid exhibited a highly positive correlation with FW = fruit weight (*r* = 0.898), LL = leaf length (*r* = 0.973), LW = leaf width (*r* = 0.985), and total fruit yield kg per treatment (*r* = 0.906). Among all the studies, very similar values of correlation were recorded.

**Table 3:** Pearson's correlation coefficient of plant vegetative growth factors, biochemical parameter, yield and its components, phenolic compound, and ascorbic acid contents of bell pepper (*Capsicum annuum* L.) foliar sprayed with cyanobacteria (*Roholtiella* sp.) HVPE and WRB under hydroponic systems.

**Traits**	**SH**	**NL**	**SI**	**SD**	**FL**	**FW**	**LL**	**LW**	**NFP**	**FWP**	**TFY**	**TSS**	**PA**	**AS**
														
SH	1													
NL	0.981^*^	1												
SI	0.954^*^	0.918^*^	1											
SD	0.883^*^	0.885^*^	0.881^*^	1										
FL	0.897^*^	0.795	0.902^*^	0.771	1									
FW	0.908^*^	0.921^*^	0.908^*^	0.991^*^	0.763	1								
LL	0.959^*^	0.984^*^	0.905^*^	0.803	0.754	0.857^*^	1							
LW	0.959^*^	0.989^*^	0.892^*^	0.814^*^	0.744	0.864^*^	0.998^*^	1						
NFP	0.506	0.402	0.699	0.63	0.697	0.613	0.344	0.317	1					
FWP	0.99^*^	0.972^*^	0.916^*^	0.817^*^	0.878^*^	0.846^*^	0.963^*^	0.964^*^	0.402	1				
TFY	0.981^*^	0.944^*^	0.945^*^	0.809	0.915^*^	0.832^*^	0.94^*^	0.934^*^	0.481	0.985^*^	1			
TSS	0.925^*^	0.94^*^	0.874^*^	0.874^*^	0.75	0.884^*^	0.916^*^	0.925^*^	0.351	0.915^*^	0.921^*^	1		
PA	0.983^*^	0.981^*^	0.946^*^	0.82^*^	0.841^*^	0.866^*^	0.989^*^	0.983^*^	0.447	0.983^*^	0.972^*^	0.906^*^	1	
AS	0.947^*^	0.989^*^	0.858^*^	0.862^*^	0.712	0.898^*^	0.973^*^	0.985^*^	0.282	0.946^*^	0.906^*^	0.949^*^	0.95^*^	1

*SH, shoot height; NL, number of leaves; SI, SPAD index; SD, shoot diameter; FL, fruit length; FW, fruit weight; LL, leaf length; LW, leaf weight; NFP, number of fruits per plant; FWP, fruit weight per plant; TFY, total fruit yield kg per treatment; TSS, total soluble solids; PA, phenolic compound; AS, ascorbic acid*.

* *Correlation is significant at p < 0.05 (5% significant level)*.

## Discussion

Bell pepper cultivated in a greenhouse drip hydroponics technique using *Roholtiella* sp. HVPE and WRB as the source of nutrients was ascertained in this study to give significant results in terms of vegetative growth parameters, chlorophyll content (SPAD index/value), yield components, total yield, and fruit quality commensurate with those achievable with prevailing hydroponic systems using organic fertilizer. Notably, from our results, (a) bell pepper treated with HVPE and WRB at various concentrations exhibited better growth parameters compared to bell pepper foliar treated without HVPE and WRB. This is due to the presence of important bioactive compounds, phytohormones, phycobiliproteins, and growth enzymes in them. (b) Also, plants treated with HVPE and WRB produced larger fruits in size compared to fruits produced from the bell pepper treated with HVPE and WRB because of the substantial availability of chlorine ions in both HVPE and WRB as well as other mineral nutrients, which facilitates the higher accumulation of chlorine ions in the bell pepper fruits. (c) Plants treated with HVPE and WRB produced high-quality fruits than fruits foliar sprayed without HVPE and WRB. All these results are due to the variation of prevailing activities as the plants respond to HVPE and WRB compared to the control group.

In this study and from our findings, the HVPE and WRB are shown to significantly increase the vegetative growth parameters of bell pepper, i.e., shoot length, the number of leaves, plant leaf length, plant leaf width, and the diameter of the shoot. These results are likely to be connected with the presence of metabolite substances, namely, phytohormones, phycobiliproteins, indoles, peptides, amino acids, and polysaccharides, in *Roholtella* sp. HVPE and WRB are known to be major players as regulators of plant growth. This finding is supported by a previous study conducted by Lee et al. (2008). The previous investigation has equally shown that algae crude extracts and biomass, as well as compounds that are purified, can cause strong physiological responses in plants and growth parameters, namely, shoot and root weight (Michalak et al., 2016; Zheng et al., 2016; Yakhin et al., 2017; Ertani et al., 2018; Hamed et al., 2018), which falls in line with our findings. It was recorded by several studies that microalgae extracts, such as polysaccharides, enhanced crop productivity (Vacca et al., 2004; Obertello et al., 2010; Ding et al., 2018; Jagodzik et al., 2018), and this is likely responsible for the enhancing potential of HVPE and WRB as shown in our results. Interestingly, our findings on the vegetative parameters were consistent with previous findings which proved that organic material had a beneficial effect on crops such as rice and soybean (Wang et al., 2012, 2016; Liu et al., 2022). Also, the impacts of HVPE and WRB were felt on the chlorophyll content in bell pepper plants through the entire growth stage. In this study, it was observed that the chlorophyll content (SPAD value) of bell peppers treated with HVPE and WRB was higher than the untreated with HVPE and WRB, irrespective of the concentration levels. This established that the availability of HVPE and WRB could improve the supplied nutrients through the leaf stomata, resulting in increased plants' chlorophyll content. Our results are in line with previous results reported by Bello et al. (2021a) on bell pepper seedlings, a study on sugar beet by Enan et al. (2016), and the evaluation of tomatoes by El-Sayed et al. (2018), and El-Eslamboly et al. (2019). which proved that the availability of biofertilizer improved the chlorophyll content of plants (Enan et al., 2016; El-Sayed et al., 2018; El-Eslamboly et al., 2019).

Furthermore, as per the results in yield components and yield itself, bell pepper plants treated with HVPE and WRB have shown significantly different positive changes in the number of fruits per plant and fresh weight in gram per fruit compared with untreated bell pepper plants. Naturally, the number of fruits per plant and fresh weight in grams per fruit as the yield components are the function of the total fruit yield, meaning that when these components increase, the yield will increase. Though this phenomenon could change when they are not fully independent, that is, when one component increases and the other decreases, the yield could be affected. Therefore, the obtained results may be connected to the impacts of several growth constituents in the composition of HVPE and WRB, namely, phytohormones, fatty acids, and amino acids, coupled with the potentials of blue-green algae (cyanobacteria) to reserve a quite large amount of mineral nutrients (El-Eslamboly et al., 2019). Similarly, from our results, the ability of HVPE and WRB to positively enhance the photosynthetic pigments has greatly influenced the carbohydrate storage at the optimal level, which invariably leads to the higher yield and its respective components. All these results have been supported by the findings reported by El-Eslamboly et al. (2019) on the impacts of algae extract on the yield characteristics and total yield of cucumber. Our results are supported by the findings from a study conducted by Ahmed and Shalaby (2012) that reported the large composition of amino acids together with the huge amounts of fatty acids as well as polypeptide hormones in algae extract, which play a major role in the improvement of the vegetative growth, yield and fruit quality of cucumbers.

The data reported in this study indicated that HVPE and WRB foliar sprayed bell pepper plants showed a significant increase in all the fruit constituents at different concentrations of HVPE and WRB, which may be a result of their mineral composition such as nitrate (nitrogen), phosphorus, sodium, and potassium (Table 2), as well as the antioxidant and enhancer potential of algae generally. There is a significant increase in the accumulation of TSS, ascorbic acid, and phenolics as the concentration of HVPE and WRB increases. The total phenolic contents for the raw bell pepper fall in the range of those previously reported (1152.8–1344.8 μg GAE g-1 fresh tissue or fresh weight) by Turkmen et al. (2005) and de Jesús Ornelas-Paz et al. (2010). In support of our results on the phenolic content, findings from several studies have shown that there could be an accumulation of a reasonable amount of phenolic compounds in microalgae and cyanobacteria extract, leading to increasing crop production (Turkmen et al., 2005; de Jesús Ornelas-Paz et al., 2010). In a study conducted by Haoujar et al. (2019), a remarkable amount of phenolic content was found in the *P. tricornutum, Nannochloris* sp., and *T. suecica* extract (Haoujar et al., 2019). These results are in support of our findings that the phenolic content in a bell pepper is greatly enhanced by the HVPE and WRB, respectively. It is important to establish that the phenolic compound accumulation could be greatly influenced by the growth parameters, namely, temperature, nutrient availability, and applicable stress, associated with the strain coupled with the prevailing methods employed in extraction. Generally, all these significant increases in the parameters are a result of the availability of bioactive compounds and the enhancing potential of *Roholtiella* sp. HVPE and WRB, respectively. Fresh bell peppers are a good source of ascorbic acid and they are so rich in this content irrespective of their pigments (green, red, or orange) ranging from 76 to 243 mg 100 g^−1^ fresh weight (Howard et al., 1994; Deepa et al., 2007). The positive contributions of phenolic compounds and ascorbic acid on bell pepper vegetative growth parameters, total soluble solids, and yield were established by their strong positive correlations with shoot height, the number of leaves, length and width of leaves, fruit weight, total fruit yield, and total soluble solids (Table 3).

Importantly, with the assessment of all the trials, similar vegetative growth parameters, yield components, total yield, and fruit quality between HVPE and WRB treatments were evaluated. This is an indication that both have the enhanced potential to improve crop productivity. These results were duly supported by several similar studies conducted to investigate the effect of biofertilizer/biostimulants on crop production. Despite the positive performances of HVPE and WRB, there are still slight differences between both in terms of the outcomes on the parameters examined. Based on our findings, HVPE exhibited better results than WRB, and this may be connected to the perfect or total dissolution of bioactive nutrients in the extract. This study shows that the slight variation in the composition of nutrients influenced our results. For instance, the composition of chlorine ions in HVPE is greater than that of WBR, which could have been responsible for the earlier producing better fruit yield, particularly in size. This finding is in line with previous reports by Kechasov et al. (2021) which confirm that high chlorine ion in organic fertigation nutrient has a great influence on fruit large size and, subsequently, a better yield based on the study conducted on the effects of organic nutrients on tomato plants. However, both have proved an enhanced potential either as an extract or resuspended biomass (Kechasov et al., 2021).

Finally, in this study, the foliar application of HVPE and WRB as biofertilizers was seen as a probable alternative to chemical fertilizers. The positive results obtained are a piece of evidence that the elemental composition of both organic nutrient sources is enough to sustain the production of long-term vegetables like bell pepper without the need for a supplement with essential micronutrients (Table 2). To the best of our understanding, our study is the first in Qatar to investigate the effects of *Roholtiella* sp. HVPE and WRB simultaneously in hydroponic agriculture.

## Conclusion

This study clearly demonstrated the foliar application of cyanobacteria (*Roholtiella* sp.) HVPE and WRB at various concentrations positively affected the bell pepper growth parameters, fruits production yields, and quality. Collectively, our results from this study suggest that the investigated cyanobacteria (*Roholtiella* sp.) isolated locally can serve as a basis for enhancing vegetative growth characteristics, fruit components, yield, and fruit quality of bell peppers as a biofertilizer candidate. Based on this finding, bell pepper could be considered as a template crop, and similar results could be expected on similar crops if *Roholtiella* sp. extract (HVPE) and biomass (WRB) are used to promote the high production of vegetable and non-vegetable plants.

## Data Availability Statement

The raw data supporting the conclusions of this article will be made available by the authors, without undue reservation.

## Author Contributions

AB: conceptualization, methodology and data acquisition, formal analysis, investigation, writing—original draft, and writing—review and editing. IS: conceptualization, writing—review and editing, and resources. TA: conceptualization, methodology, writing—review and editing, and formal analysis. HH: conceptualization and writing—review and editing. MC: methodology and data acquisition and formal analysis. RB-H: conceptualization, writing—review and editing, supervision, funding acquisition, and resources. All authors have read and agreed to the published version of the manuscript.

## Funding

This study was funded by the Graduate Student Grant (QUST-1-CAS-2020-10) provided by Qatar University, the NPRP8 project, and Qatar University IDC funds of RB-H. The statements made herein are solely the responsibility of the authors.

## Conflict of Interest

The authors declare that the research was conducted in the absence of any commercial or financial relationships that could be construed as a potential conflict of interest.

## Publisher's Note

All claims expressed in this article are solely those of the authors and do not necessarily represent those of their affiliated organizations, or those of the publisher, the editors and the reviewers. Any product that may be evaluated in this article, or claim that may be made by its manufacturer, is not guaranteed or endorsed by the publisher.
